# First isolation and genomic insights of *Bartonella henselae* from domestic cats and dogs in Costa Rica

**DOI:** 10.3389/fmicb.2026.1870135

**Published:** 2026-07-29

**Authors:** Gabriela González-Espinoza, José Arturo Molina-Mora, Catalina Chaves-Leon, Karen Solis-Jiménez, Grettel Vega-Álvarez, Ana Mariel Zúniga-Pereira, Carlos Chacón-Díaz, Norman Rojas-Campos

**Affiliations:** Facultad de Microbiología, Centro de Investigación en Enfermedades Tropicales (CIET), Universidad de Costa Rica, San José, Costa Rica

**Keywords:** *Bartonella henselae*, *Bartonella* isolation, companion animals, Costa Rica, pan-genome analysis, vector-borne disease, zoonosis

## Abstract

*Bartonella* spp. are vector-borne zoonotic pathogens responsible for cat-scratch disease and several other human disease manifestations. Previous studies in Costa Rica have reported *Bartonella* DNA in ectoparasites and bats; however, data from companion animals remain limited. Here, we investigated the occurrence of *Bartonella* spp. in domestic cats and dogs and characterized the circulating strains using molecular, culture-based, and genomic approaches. Blood samples were collected from 152 domestic cats and 188 domestic dogs from different Costa Rican regions. Conventional polymerase chain reaction (PCR) assays targeting citrate synthase (*gltA*) and the beta subunit of bacterial RNA polymerase (*rpoB*) were used to detect *Bartonella* spp. DNA was detected in 44% of the cats and 34% of the dogs. Partial *gltA* sequencing identified *B. henselae*, *B. rochalimae,* and *B. clarridgeiae*. Four *B. henselae* isolates recovered from feline blood were subjected to whole-genome sequencing. Comparative pan-genome and multilocus sequence typing (MLST) analyses identified sequence types ST1 and ST5 circulating in Costa Rica. These findings provide the first genomic characterization of *Bartonella* spp. from companion animals in Costa Rica and expand the current knowledge of the genetic diversity and epidemiology of *Bartonella* in Central America.

## Introduction

The genus *Bartonella* comprises Gram-negative, fastidious, facultative intracellular Alphaproteobacteria transmitted primarily by hematophagous arthropods, including fleas, ticks, and lice (Sofer et al., 2015). The genus currently includes approximately 40 recognized species that infect a wide range of mammalian hosts (Huang et al., 2019; Rao et al., 2021; He et al., 2025). Fifteen species have been associated with zoonotic infections in humans, and several are of public health relevance owing to their global distribution and capacity to infect multiple hosts (Dehio, 2004; Breitschwerdt et al., 2010; Regier et al., 2016).

*Bartonella henselae*, *Bartonella quintana*, and *Bartonella bacilliformis* account for the most reported human infections, whereas *Bartonella koehlerae*, *Bartonella clarridgeiae*, *Bartonella alsatica*, and *Bartonella vinsonii subsp. berkhoffii* are reported less frequently (Avidor et al., 2004; Chomel et al., 2004; Logan et al., 2019). Human bartonellosis ranges from self-limiting cat-scratch disease to severe manifestations, such as bacillary angiomatosis, endocarditis, and neurological complications, particularly in immunocompromised individuals. Humans usually acquire *B. henselae* through scratches or bites from infected cats, whereas direct arthropod-associated transmission is less frequent (Chomel et al., 1996; Oskouizadeh et al., 2010).

Domestic cats (*Felis catus*) are the principal reservoir hosts of *B. henselae* and also serve as reservoirs for *B. clarridgeiae* and *B. koehlerae* (Dehio, 2004). Infected cats are generally asymptomatic but can develop persistent or recurrent intraerythrocytic bacteremia, although the long-term clinical significance of infection in cats remains unclear (Avidor et al., 2004; Chomel et al., 2004, 2006; Müller et al., 2017; Raimundo et al., 2019).

Domestic dogs (*Canis familiaris*) are considered the main reservoir hosts of *B. vinsonii subsp. berkhoffii* and can also be infected with *B. henselae*, *B. koehlerae*, *B. clarridgeiae*, *B. elizabethae*, *B. rochalimae*, and *B. washoensis* (Breitschwerdt et al., 2010; Álvarez-Fernández et al., 2018; Ernst et al., 2020). Dogs are generally regarded as accidental rather than reservoir hosts for *B. henselae*, although this species is among the most frequently reported *Bartonella* spp. in canine infections (Chomel et al., 2009; Breitschwerdt et al., 2010). The clinical manifestations in dogs may resemble those described in humans and include endocarditis, fever, uveitis, lymphadenopathy, arthritis, vasculitis, and neurological disorders (Álvarez-Fernández et al., 2018).

The distribution of *Bartonella* spp. in cats is influenced by environmental and climatic conditions that affect flea abundance, particularly that of *Ctenocephalides felis*, the principal vector of *B. henselae*. A lower prevalence is generally observed in colder regions, where reduced flea populations may limit transmission among feline hosts (Chomel et al., 2006).

In the Americas, *Bartonella* spp. infections have been documented in cats in Argentina (Cicuttin et al., 2014), Chile (Müller et al., 2017), Paraguay (Sepúlveda-García et al., 2022), Brazil (Braga et al., 2015; Furquim et al., 2021; Dias et al., 2023; Das Neves et al., 2025), Colombia (Brenner et al., 2013), Guatemala (Braga et al., 2015), and Mexico (Tobar et al., 2020). *Bartonella* spp. have also been detected in cat-associated fleas in Argentina (Urdapilleta et al., 2020), Chile (Müller et al., 2017), Peru (Rizzo et al., 2015), Costa Rica (Rojas et al., 2015), Guatemala (Braga et al., 2015), and Mexico (Tobar et al., 2020). These studies identified several zoonotic *Bartonella* species, including *B. henselae*, *B. clarridgeiae*, and *B. koehlerae*. *Bartonella* infections have also been documented in dogs across the Americas, particularly among free-roaming animals in tropical regions (Brenner et al., 2013; Tobar et al., 2020). Given that dogs can be infected with multiple *Bartonella* species and may develop clinical signs comparable to those of humans, they are considered useful sentinels for *Bartonella* exposure and circulation in shared environments (Chomel et al., 2006).

Despite evidence of the circulation of *Bartonella* in mammals, genomic information from Central America remains limited. In Guatemala, *B. henselae* and *B. clarridgeiae* have been detected in cats and their fleas (Bai et al., 2015). In Costa Rica, molecular studies have identified *B. vinsonii subsp. berkhoffii*, *B. rochalimae*, *B. henselae*, and *B. clarridgeiae* in *Ctenocephalides felis* and *Pulex simulans* fleas collected from domestic cats (Rojas et al., 2015). *Bartonella* DNA has also been detected in bats and their ectoparasites, suggesting complex transmission cycles and circulation of previously unrecognized species in wildlife (Mitchell et al., 2022). However, genomic data on *Bartonella* spp. circulating in companion animals in Costa Rica are scarce. Therefore, this study investigated the occurrence and genomic characteristics of *Bartonella* spp. in domestic cats and dogs sampled from different regions of Costa Rica.

## Materials and methods

### Sample collection and ethical approval

Dogs and cats were sampled between 2014 and 2018 during spay-neuter campaigns conducted by veterinarians in five regions of Costa Rica. Sampling was based on animal availability and owner consent and was not designed to provide a nationally representative sample of the country’s cat and dog populations. Age, sex, and breed were not used as inclusion or exclusion criteria. Ethylenediaminetetraacetic acid (EDTA)-anticoagulated blood was processed immediately for bacterial isolation, and the remaining sample volume was stored at −70 °C until DNA extraction. Animal protocols were reviewed and approved by the Comité Institucional para el Cuido y Uso de los Animales of the Universidad de Costa Rica (CICUA-39-14) and complied with Costa Rican Animal Welfare Law No. 7451 (Ley de Bienestar de los Animales).

### Isolation of *Bartonella* from blood

Immediately after blood collection, 400 μL of whole blood from dogs and cats was inoculated dropwise onto chocolate agar. The plates were incubated under high-humidity, capnophilic conditions (approximately 5% CO_2_) at 37 °C for at least 15 days. Bacterial growth was monitored daily. Colonies were presumptively identified as *Bartonella* based on their slow growth and small, grayish morphology. Colonies suspected to be *Bartonella* spp. were subcultured on fresh agar plates and incubated under the same conditions until sufficient growth was observed. Pure suspected colonies were subjected to Gram staining and specific PCR targeting the *Bartonella gltA* and *rpoB* genes. Once confirmed by PCR, pure cultures were cryopreserved at −80 °C in liquid nitrogen in Brain Heart Infusion broth containing 20% glycerol.

### Blood DNA extraction

Genomic DNA from the blood samples was extracted using the DNeasy Blood and Tissue Kit (QIAGEN GmbH, Hilden, Germany), according to the manufacturer’s protocol. Bacterial DNA from cultured isolates was extracted using the Wizard Genomic DNA Purification Kit (Promega) according to the manufacturer’s instructions.

### Molecular detection of *Bartonella* DNA

During spay-neuter campaigns, veterinarians collected blood samples while the animals were under anesthesia. Brachial venous blood was collected into EDTA tubes and immediately processed for bacterial isolation on agar plates under a capnophilic atmosphere. The remaining blood was stored at −80 °C until DNA extraction. Specific PCR assays were performed to detect *Bartonella* spp. DNA in domestic animals. Two molecular targets were used: the *gltA* gene encoding citrate synthase (750 bp), amplified with primers CS443f (5-′GCTATGTCTGCATTCTATCA-3′) and CS1210r (5-′GATCYTCAATCATTTCTTTCCA-3′) (Birtles and Raoult, 1996; Billeter et al., 2011) and a 795-bp fragment of the *rpoB* gene encoding the RNA polymerase beta-subunit, amplified with primers 1400F (5-′CGCATTGGCTTACTTCGTATG-3′) and 2300R (5-′GTAGACTGATTAGAACGCTG-3′) (Renesto et al., 2001). The *gltA* amplification protocol consisted of 2 min at 94 °C, followed by 45 cycles of 30 s at 94 °C, 60 s at 52 °C, and 90 s at 72 °C, and a final extension of 10 min at 72 °C. The *rpoB* amplification protocol consisted of 2 min at 95 °C, followed by 40 cycles of 20 s at 95 °C, 30 s at 51 °C, 160 s at 72 °C, and a final extension of 10 min at 72 °C.

Only the *gltA*-positive PCR products were sequenced and used for phylogenetic analysis. All positive *gltA* amplicons were purified using the QIAquick Gel Extraction Kit (QIAGEN GmbH, Hilden, Germany) and sequenced in both directions using Macrogen Korea (Seoul, Republic of Korea) amplification primers. Forward and reverse chromatograms were reviewed and edited manually. Consensus sequences were compared with GenBank sequences using National Center for Biotechnology Information (NCBI) Basic Local Alignment Search Tool (BLAST), and the edited sequences were aligned with homologous sequences retrieved from GenBank.

### Phylogenetic analysis of *gltA* sequences

For phylogenetic analysis based on *gltA*, nucleotide sequences were aligned and analyzed using DNA Sequence Polymorphism version 6 (DnaSP v6; Universitat de Barcelona, Barcelona, Spain)^1^ to estimate genetic diversity parameters, including nucleotide diversity (pi), haplotype diversity (Hd), number of haplotypes (h), and haplotype distribution among samples (Rozas et al., 2017). Haplotype information was subsequently used to reduce redundancy among identical sequences and to characterize the intraspecific genetic diversity of *Bartonella* spp. detected in this study.

*gltA* sequences were aligned and compared among the isolates and with sequences from other *Bartonella* species using Molecular Evolutionary Genetics Analysis (MEGA12) version 12.0 (Kumar et al., 2016). Phylogenetic reconstruction was performed using the maximum-likelihood method implemented in MEGA12. Before tree inference, the best-fitting nucleotide substitution model was determined using the Model Selection function in MEGA12. The models were compared using the Bayesian Information Criterion (BIC), and the Kimura 2-parameter model with gamma-distributed rate variation among sites (K2 + G) was selected as the optimal model (BIC = 5,986.150). The final alignment included 19 representative sequences and 554 nucleotide positions. *Brucella abortus* was used as the outgroup. The phylogenetic tree generated in Newick format was visualized using the Interactive Tree of Life (iTOL) v5.^2^

### Morphological characterization of *Bartonella* isolates

Gram staining was performed for all isolated colonies. Texas Red fluorescent labeling and immunostaining with polyclonal antibodies against *Bartonella* were performed. Briefly, bacteria from fresh cultures were washed with phosphate-buffered saline (PBS), pelleted, and stained with Texas Red solution (stock concentration, 1 mg/mL; Invitrogen). Bacteria were incubated for 20 min at room temperature and washed three times in PBS to remove the unbound dye. For immunostaining, in-house mouse polyclonal antibodies against *Bartonella henselae* were used to screen *Bartonella* colonies before sequencing confirmation. Alexa Fluor 488-conjugated goat anti-mouse antibody (Life Technologies) was used as the secondary antibody. Images were acquired using a Nikon C-HGFI Intensilight (Nikon Instruments Inc., NY, USA) precentered fiber illuminator at 40x magnification and processed using ImageJ software (National Institutes of Health, MD, USA).

### MLST analysis

*Bartonella henselae* sequence types (STs) were identified using the *B. henselae* MLST database available through PubMLST.^3^ Allelic profiles obtained from the genome sequences were compared with the reference MLST scheme to assign STs. The identified STs were then analyzed using GrapeTree through PubMLST to generate a minimum-spanning tree and assess the genetic relationships between Costa Rican isolates and the American and global *B. henselae* population structure (Zhou et al., 2018). A total of 37 STs currently available in the database were included in the analysis.

### Whole-genome sequencing

Sample preparation: Genomic DNA was extracted using the Wizard Genomic DNA Purification Kit (Promega) according to the manufacturer’s protocol. DNA quality and concentration were assessed using a NanoDrop spectrophotometer, and DNA integrity was confirmed by agarose gel electrophoresis.

Library preparation and sequencing: Whole-genome DNA was sequenced to an approximate output of 200 Mbp (1.33 million reads) using Illumina technology (Illumina Inc., CA, USA) at SeqCenter (Pittsburgh, PA, United States). Briefly, sample libraries were prepared using the tagmentation- and PCR-based Illumina DNA Prep kit and custom Integrated DNA Technologies (IDT) 10-bp unique dual indices, with a target insert size of 280 bp. No additional DNA fragmentation or size-selection steps were performed. Illumina sequencing was performed on a NovaSeq X Plus sequencer, producing 2 × 151-bp reads. Demultiplexing, quality control, and adapter trimming were performed using the bcl-converter v3.9.3.

### Genome assembly and annotation

Bioinformatic analyses were performed on the High-Performance Computing–Universidad de Costa Rica (HPC–UCR) computational cluster (Universidad de Costa Rica, https://hpc.ucr.ac.cr/) using custom scripts described previously (Molina-Mora et al., 2020, 2025). Raw reads were evaluated using FastQC v0.12.1 (Andrews, 2010) before and after trimming. Low-quality reads (Q < 30) and adapters were removed using Trimmomatic v0.38 (Bolger et al., 2014).

*De novo* genome assembly was performed using Unicycler v0.5.1 (Wick et al., 2017), with a minimum contig length of 1,000 bp. Genome assembly quality was evaluated using the 3C criterion (Molina-Mora and García, 2020; Molina-Mora et al., 2020). Gene prediction and structural annotation were performed using Prokka v1.14.6 (Seemann, 2014). Functional annotation focused on multilocus sequence typing using MLST v2.0^4^ within the PathogenWatch platform.^5^ Virulence-associated genes were screened using the Virulence Factor Database (VFDB).^6^

### Comparative genomic analysis by a pan-genome approach

All available *Bartonella henselae* genome sequences (*n* = 55; FASTA format) were retrieved from NCBI Genomes.^7^ The sequences were structurally annotated using Prokka, as described above. The resulting General Feature Format (GFF) annotation files were used for phylogenetic reconstruction through pan-genome analysis based on gene presence/absence using Roary v3.12.0 (Page et al., 2015). The phylogenetic tree generated in the Newick format was visualized using the Interactive Tree of Life (iTOL) v5 (see text Footnote 2).

### Statistical analysis

Associations between categorical variables were evaluated using Pearson’s chi-square tests. This test was used to assess differences in the frequency of *Bartonella* detection among the evaluated groups and study localities. As some regions had small sample sizes and at least one zero count, we combined the two Limón subregions into a single category and the two Puntarenas subregions into a single category before performing the analysis. Statistical analyses were performed using GraphPad Prism (GraphPad Software LLC, MA, USA) version 10, and the results were considered statistically significant at *p* < 0.05.

## Results

### Detection of *Bartonella* spp. in domestic animals

To assess the occurrence and potential circulation of *Bartonella* spp. in companion animals in Costa Rica, 340 animals, including 152 domestic cats and 188 domestic dogs, were tested. Age, sex, and breed were not used as inclusion or exclusion criteria. Blood samples were collected from animals enrolled in voluntary spay/neuter campaigns conducted between 2014 and 2018 from diverse communities and management settings. Although this approach facilitated sample collection, the study population may differ from the broader regional animal population in terms of geographic origin, access to veterinary services, owner participation, and animal management practices.

**Figure 1:**
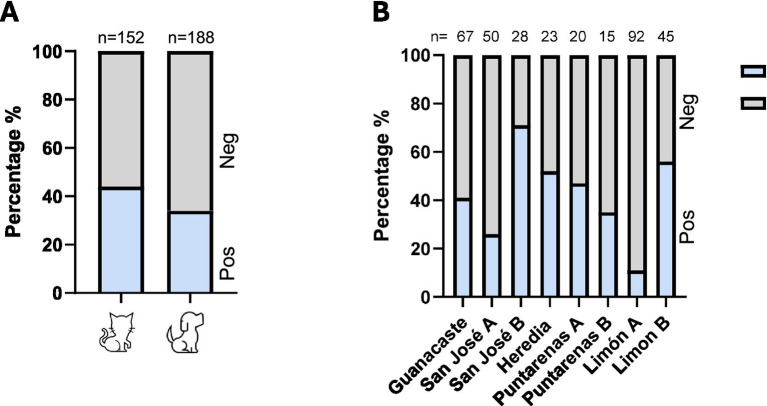
Detection of *Bartonella* spp. in domestic cats and dogs from Costa Rica. **(A)** Percentage of *Bartonella* PCR-positive and PCR-negative animals based on *gltA, rpoB,* or both targets. No significant difference in *Bartonella* detection was observed between the animal species (Fisher’s exact test, *p* = 0.1918). **(B)** Percentage of *Bartonella* PCR-positive and PCR-negative animals based on *gltA*, *rpoB*, or both targets across the sampling regions in Costa Rica. The detection frequency was reported as the percentage of samples that tested positive for *Bartonella*. Numbers above the bars indicate the total number of animals tested. A chi-square test of independence showed significant differences in *Bartonella* detection among regions χ^2^ (15) = 91.59, *p* < 0.001.

*Bartonella* DNA was screened by PCR targeting the citrate synthase (*gltA*) and beta-subunit of bacterial RNA polymerase (*rpoB*) genes, which provide discriminatory power for distinguishing among *Bartonella* spp. (Scola et al., 2003). Overall, 39% of the animals (131/340) tested positive for *Bartonella* DNA by *gltA* and/or *rpoB* PCR, including 44% of cats (67/152) and 34% of dogs (64/188) (Figure 1A). The amplification results differed between the *gltA* and *rpoB* assays, with several animals testing positive for only one target (Table 1). As amplification success varied between PCR targets, only the *gltA* amplicons were included in subsequent sequence-based analyses. Because sampling was based on surveillance campaign availability rather than random selection, the results should not be interpreted as representative prevalence estimates for the overall cat and dog population in Costa Rica. *Bartonella* spp. positivity was significantly higher in cats than in dogs. *Bartonella* gltA DNA was detected in 67 of 152 cats (44.1%) and 60 of 184 dogs (32.6%). A significant association was observed between host species and *Bartonella* infection status (two-sided Fisher’s exact test, *p* = 0.033). Nevertheless, *Bartonella*-positive cats and dogs were detected in all five sampled provinces (San Jose, Heredia, Limon, Puntarenas, and Guanacaste; Table 1; Figure 1B). A significant association was observed between sampling location and *Bartonella* detection in cats and dogs (Pearson’s chi-square test, χ^2^ (15) = 91.59, *p* < 0.001), indicating heterogeneity in *Bartonella* occurrence among the sampled regions.

**Table 1:** Detection of *Bartonella* spp. in domestic animals from five provinces of Costa Rica based on *gltA* and *rpoB* gene amplification, 2014–2017.

Region	Location	Total animals	Positive cats and dogs (*rpoB*)	Positive (*gltA*)	Positive cats (*gltA*)	Positive dogs (*gltA*)
Limón A	Cieneguita	92	9	10	7 (33)^*^	3 (59)
Limón B	Centro	45	1	25	9 (23)	16 (22)
Guanacaste	Santa Cruz	67	3	26	5 (18)	21 (49)
San José A	Puriscal	50	6	11	5 (15)	6 (35)
San José B	Pérez Zeledón	28	20	18	4 (8)	14 (20)
Heredia	San Rafael	23	8	12	12 (23)	0 (0)
Puntarenas A	Barranca	15	5	7	7 (15)	0 (0)
Puntarenas B	Chacarita	20	7	3	3 (17)	0 (3)
TOTAL		340	59	112	52	60

* Values in parentheses indicate the total number of dogs and cats sampled from each region.

A total of 38 *gltA* fragments from the positive samples were successfully amplified and sequenced. Sequence analysis identified *B. henselae*, *B. clarridgeiae*, and *B. rochalimae* in cat and dog populations. BLAST analysis showed that 30 of 38 sequences (78.95%) shared 99.82–100% identity with *B. henselae*. These sequences were obtained from both cats and dogs, although most were from cats, which are recognized as reservoir hosts for several *Bartonella* spp. Seven sequences (18.42%) of feline origin showed 99–100% identity with *B. clarridgeiae*, whereas one dog-derived sequence (2.63%) showed 99–100% identity with *B. rochalimae*.

### Phylogenetic analysis of *gltA* sequences

For the *Bartonella* spp. *gltA* dataset (38 sequences), 554 nucleotide positions were analyzed, of which 82 were variable. Haplotype analysis identified five haplotypes (h = 5), with a haplotype diversity (Hd) of 0.4452. Haplotype 1 was the most frequent, comprising 28 Costa Rica (CR) sequences (CR1, CR3, CR4, CR6, CR7, CR8, CR11-CR22, CR24-CR28, CR30, CR32, CR33, CR36, and CR38) and corresponding to the predominant *B. henselae* genotype. Haplotype 2 included two sequences (CR2 and CR5) and represented *B. clarridgeiae*. Haplotype 3 comprised five sequences (CR9, CR29, CR34, CR35, and CR37) and corresponded to *B. clarridgeiae*. Haplotype 4 was represented by a single sequence (CR10) corresponding to *B. rochalimae*, whereas haplotype 5 included two sequences (CR23 and CR31) and represented the second *B. henselae* genotype.

**Figure 2:**
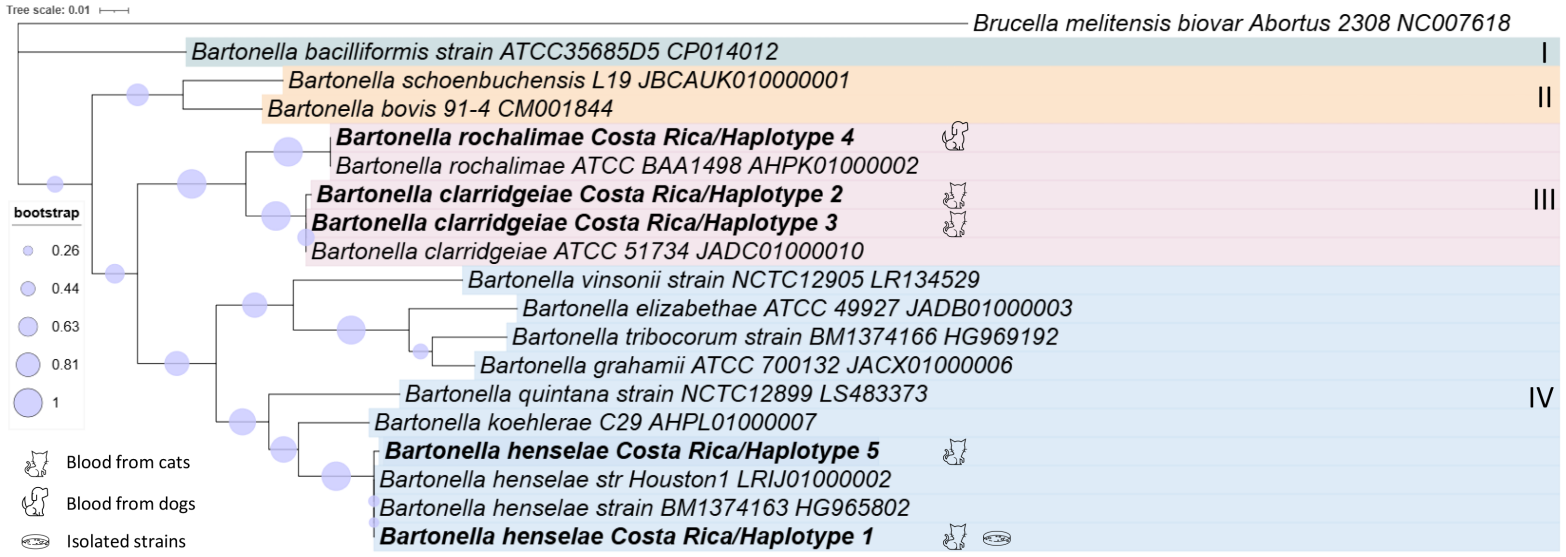
Phylogenetic relationships within *Bartonella* spp. based on a 554-bp fragment of the *gltA* gene. The tree was inferred using the maximum-likelihood method and the Kimura 2-parameter model with gamma-distributed rate variation among sites (K2 + G), with bootstrap values calculated from 1,000 replicates in MEGA version 12.0 (https://www.megasoftware.net). *Brucella abortus* was used as the outgroup. Sequences detected in this study were obtained from the blood of domestic animals and labeled with the acronym CR (Costa Rica sequence). Cat and dog icons indicate host origin, and the bacterial icon indicates the *gltA* sequences obtained from bacterial isolates.

To contextualize the evolutionary relationships among the detected *Bartonella* spp., a maximum-likelihood phylogenetic tree was constructed using representative *gltA* sequences and reference sequences from *Bartonella* species associated with animal and human diseases. The *gltA* tree (Figure 2) showed that most sequences were grouped within a *B. henselae* cluster, lineage IV (accession numbers LRIJ01000002 and HG965802), supported by a bootstrap value of 100%. The single dog-derived sequence identified as *B. rochalimae* (accession number AHPK01000002) clustered with that of the *B. rochalimae* reference strain. Seven cat-derived sequences formed a distinct cluster with the *B. clarridgeiae* reference strain (accession number JADC01000010), which was also supported by a bootstrap value of 100%. Both are within lineage III (Engel et al., 2011).

### Isolation of *Bartonella* strains in Costa Rica

**Figure 3:**
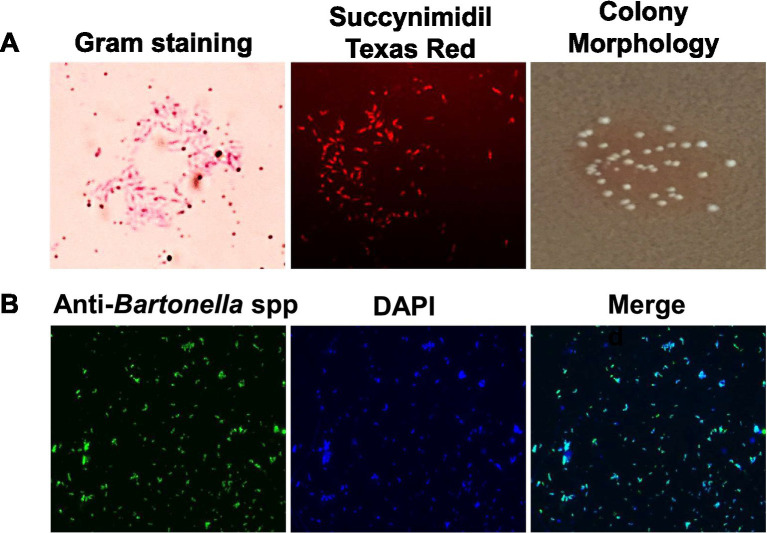
Phenotypic characterization of *Bartonella henselae* isolates from Costa Rica. **(A)** Microscopic morphology of *B. henselae* using Gram staining and Texas Red fluorescent labeling, together with macroscopic colony morphology on chocolate agar. **(B)** Immunolabeling of *B. henselae* with an anti-*Bartonella* mouse antibody.

Four isolates (CR8, CR17, CR18, and CR38; 1.7% of all samples) were obtained from feline blood samples. Colonies with a morphology consistent with *Bartonella* were observed on chocolate agar between 6 and 10 days after inoculation; colonies were slow-growing, grayish, and very small (Figure 3). Gram staining and Texas Red fluorescent labeling revealed Gram-negative coccobacilli, consistent with previously described *Bartonella* phenotypes. Immunostaining with anti-*Bartonella* polyclonal antibodies yielded a positive signal, further supporting the identity of the isolates. The isolates were confirmed to be *B. henselae* by *gltA* analysis and subsequent whole-genome comparative analyses.

### Pan-genome analysis using complete genome sequences identifies *Bartonella henselae* MLST profiles

**Figure 4:**
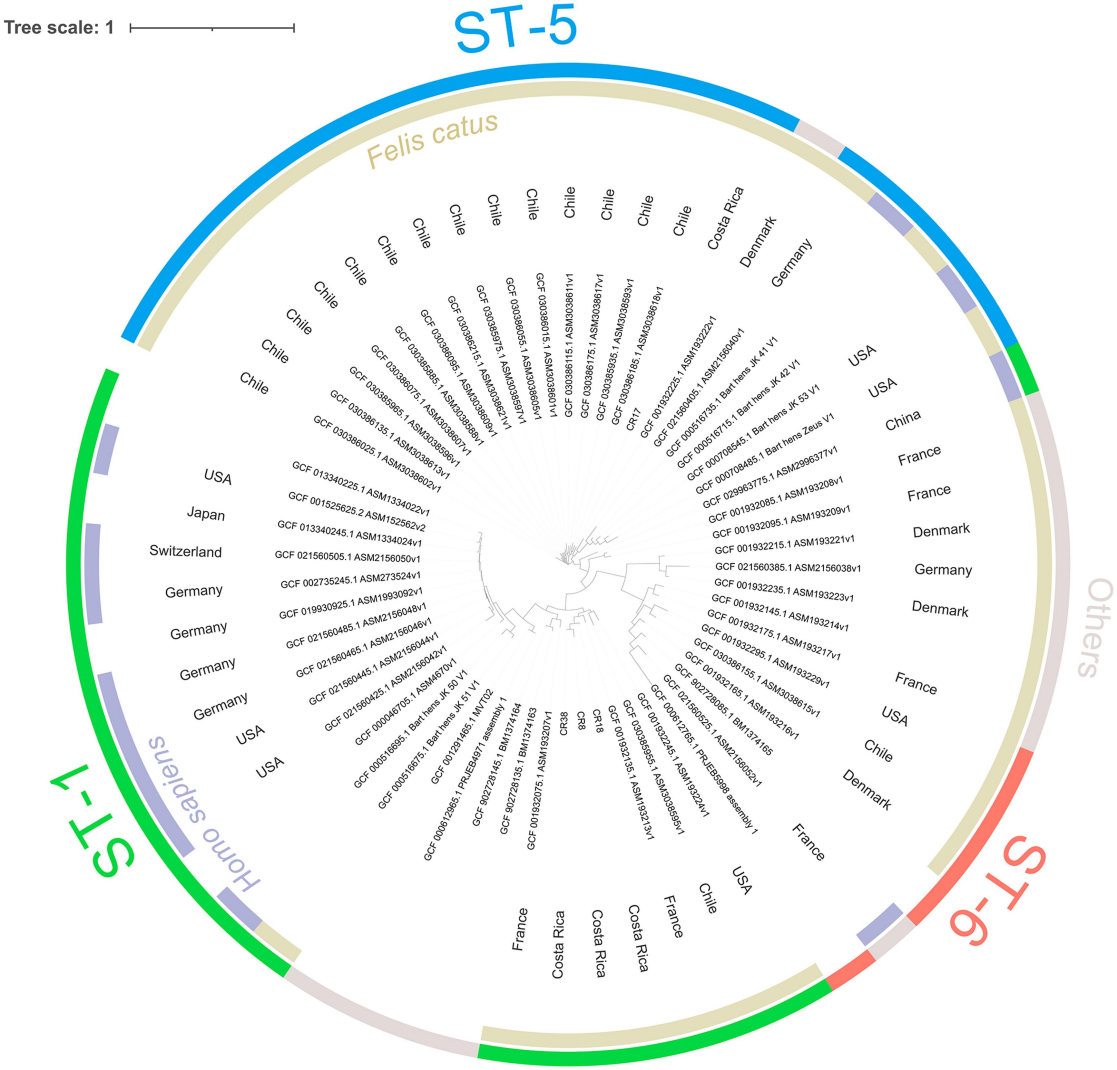
Comparative genomic analysis of Costa Rican *Bartonella henselae* strains. A pan-genome strategy was used to compare complete genomes and identify gene content patterns associated with multilocus sequence typing (MLST) profiles. Sequence-type groups with fewer than four strains are shown in gray, whereas groups comprising five or more strains are shown in distinct colors. CR17 is clustered within ST5, whereas CR8, CR18, and CR38 are clustered within ST1.

**Figure 5:**
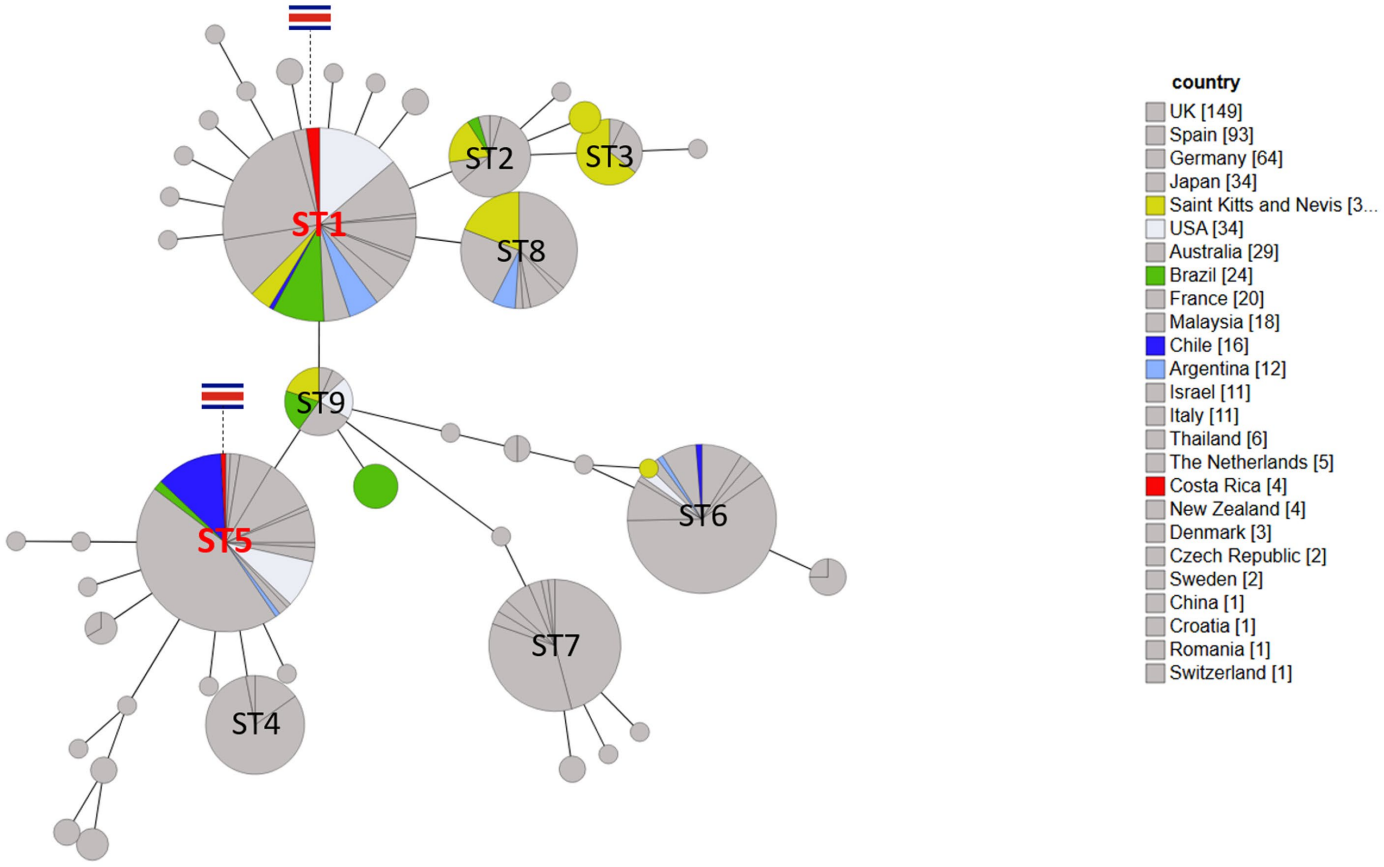
Global and population structure of *Bartonella henselae* in the Americas, highlighting the placement of Costa Rican sequence types in a minimum-spanning tree based on MLST profiles. Minimum-spanning tree generated with GrapeTree from MLST profiles of the 37 *Bartonella henselae* sequence types (STs) currently available in the PubMLST database. Nodes represent individual sequence types (STs) and are colored according to the country in which each ST has been detected. The node size is proportional to the number of isolates belonging to each ST. Only the largest clusters were labeled. Non-American countries are labeled in gray. The Costa Rican isolates characterized in this study are indicated in red, and the Costa Rican flag includes CR17 (ST5), CR8, CR18, and CR38 (ST1). The number of isolates per country is shown in parentheses.

After whole-genome sequencing, the genomes were assembled and annotated. A comparative genomic strategy was used to assess the diversity among the Costa Rican isolates (CR8, CR17, CR18, and CR38) and 54 publicly available *B. henselae* genomes from several geographic origins, including Chile (*n* = 16), the United States (*n* = 7), France (*n* = 6), Germany (*n* = 6), Denmark (*n* = 4), China (*n* = 1), Japan (*n* = 1), Switzerland (*n* = 1), and unknown locations (*n* = 12). Pan-genome analysis identified 2,821 genes across all strains, of which 1,085 (38.5%) belonged to the core genome (present in >99% of the strains). PathogenWatch analysis provided MLST profiles for all genomes. Nine distinct MLST profiles were identified. Gene presence/absence patterns distributed the strains into three main clusters that were broadly consistent with the MLST profiles. The CR17 isolate clustered within ST5, whereas the CR8, CR18, and CR38 isolates clustered within ST1. CR8 had an incomplete *batR* locus (broken by the contig end); it only missed the last 7 bp and was otherwise identical to *batR*-1. A provisional designation of *batR*-1 was assigned to this isolate, allowing sequence type (ST) to be determined. Isolates from different countries and host species were distributed throughout the phylogeny, with no obvious clustering by host or geographical origin (Figure 4). GrapeTree analysis of the 37 *B. henselae* sequence types available in the PubMLST database (Figure 5) demonstrated that ST1 and ST5 have been detected across America, supporting their widespread geographic distribution. The identification of both sequence types in Costa Rican cats indicated that globally distributed *B. henselae* lineages circulate throughout Central America. Sequence types identified in this study have been published in the PubMLST database with ID numbers 576–579.

## Discussion

The overall proportion of *Bartonella*-positive cats detected in this study (44%) is consistent with previously reported ranges of *Bartonella* bacteremia in cats (15–55%) across regions of the Americas, Oceania, Europe, Asia, and Africa (Chomel et al., 1999, 2002; Maruyama et al., 2001; Guptill et al., 2004; Banerjee et al., 2020; Furquim et al., 2021). In dogs, the global molecular prevalence of *Bartonella* spp. has been estimated to be 3.6% (Zarea et al., 2023), which is lower than the detection frequency observed here. However, differences in study design, diagnostic methods, sampled populations, and environmental conditions limit direct comparisons among studies. Variation in detection frequency among sampled sites suggests that local environmental, ecological, host-related, or vector-related factors may influence *Bartonella* transmission dynamics and that geographic proximity alone does not explain the observed heterogeneity. Since the sampling design was not intended to be representative of cat and dog populations within each province, these results should be interpreted as evidence of *Bartonella* circulation in the sampled companion animals rather than as regional prevalence estimates.

Persistent bacteremia is well documented in cats, which serve as reservoir hosts for several *Bartonella* species, whereas dogs are generally considered incidental hosts for many *Bartonella* infections. Therefore, the detection of *Bartonella* DNA in canine blood does not necessarily indicate active infection or chronic bacteremia. PCR detection alone cannot distinguish between acute and chronic infections. *Bartonella* spp. can establish persistent intravascular infections in reservoir hosts, often resulting in prolonged or relapsing bacteremia (Tahmasebi Ashtiani et al., 2024).

Most studies conducted in Central America have focused on detecting *Bartonella* in fleas collected from animals. However, successful isolation from vertebrate hosts remains uncommon because of the technical challenges associated with *Bartonella* culture. In the Eastern Mediterranean Region, the overall prevalence of *Bartonella* spp. among animals, determined using molecular and culture methods, was reported to be 11.9 and 1.7%, respectively (Tahmasebi Ashtiani et al., 2025). The discrepancy between the number of PCR-positive samples and the four isolates recovered likely reflects the greater sensitivity of PCR for detecting low bacterial loads compared with direct culture. In addition, the fastidious nature of *Bartonella* spp. and the absence of a pre-enrichment step, such as the Bartonella Alpha Proteobacteria Growth Medium (BAPGM), may have reduced the sensitivity of bacterial isolation. Consequently, some PCR-positive animals may have harbored low-level bacteremia detectable by molecular methods but not recoverable by culture (Tahmasebi Ashtiani et al., 2024). The combination of BAPGM pre-enrichment, molecular detection, and subsequent subculture on blood or chocolate agar has substantially improved the sensitivity of *Bartonella* detection and isolation (Dias et al., 2023; Das Neves et al., 2025). Despite these limitations, our findings provide important evidence of Bartonella circulation among cats and dogs in Costa Rica. Future studies incorporating larger sample sizes, broader geographic coverage, and enhanced culture methodologies are needed to better characterize the epidemiology, transmission dynamics, and clinical significance of *Bartonella* infections throughout the country.

The detection of *Bartonella* spp. in multiple geographical regions provides evidence that these pathogens circulate among companion animals in Costa Rica. A significant association was observed between sampling location and *Bartonella* detection, indicating a heterogeneous occurrence across the sampled regions. Differences in detection frequency were observed between geographically proximate locations, indicating that local ecological, environmental, vector-related, and animal management factors may influence *Bartonella* transmission. Given that this study used convenience sampling rather than a population-based design, these findings should be interpreted as differences among sampled populations rather than as estimates of regional or national prevalence. Nevertheless, the results highlight the widespread detection of *Bartonella* spp. in sampled Costa Rican cats and dogs and underscore the need for further investigations using larger sample sizes, broader geographic coverage, and detailed animal- and vector-level data.

The detection of *Bartonella* spp. in ectoparasites in Costa Rica (Rojas et al., 2015), together with the present findings, supports the circulation of these bacteria among companion animals and their associated vectors. *Bartonella henselae* was the predominant species identified in both cats and dogs, with *B. rochalimae* additionally detected in dogs *and B. clarridgeiae* detected in cats. These species have previously been found in fleas commonly associated with feline and canine hosts, including flea species that are capable of feeding on humans (Rojas et al., 2015).

The high frequency of ectoparasites reported in dogs from rural communities on the Caribbean slope of Costa Rica highlights their potential role in maintaining and transmitting vector-borne pathogens (Troyo et al., 2012). Fleas were the most common ectoparasites, with *Ctenocephalides felis* and *Pulex simulans* predominating and frequently co-occurring. Both flea species have previously been found to harbor *Bartonella* spp. DNA in Costa Rica, thereby supporting its potential epidemiological relevance in *Bartonella* transmission. The coexistence of multiple ectoparasite species in individual dogs may further increase the opportunities for pathogen circulation. Although data on ectoparasites infesting cats in these communities are currently lacking, both *C. felis* and *P. simulans* can parasitize cats and dogs. Given the close coexistence of these animals within household environments, similar patterns of ectoparasite infestation and pathogen exposure may occur in cats and warrant further investigation.

Among the detected species, *B. henselae* is a public health concern, as it is the principal causative agent of cat-scratch disease. Although most infections are mild and self-limiting, atypical manifestations have been reported in 1–20% of cases and may include severe complications such as encephalitis, retinitis, osteomyelitis, atypical pneumonia, prolonged fever, and other neurological disorders (Jurja et al., 2022). *Bartonella rochalimae* is a zoonotic species first identified in a human case of febrile illness associated with travel to South America (Eremeeva et al., 2007). Canids, including domestic dogs and wild carnivores such as coyotes, foxes, and raccoons, are considered the main reservoirs (Chomel et al., 2009; Henn et al., 2009; Álvarez-Fernández et al., 2018). Therefore, the molecular detection of *B. rochalimae* in dogs in the present study highlights the potential circulation of a pathogen of both veterinary and public health relevance. Although human infections are rare, *B. rochalimae* can cause febrile illness and bacteremia, confirming its zoonotic relevance (Chomel et al., 2009; Henn et al., 2009; Álvarez-Fernández et al., 2018). To date, no confirmed human case of bartonellosis has been documented in Costa Rica in the published literature. Nevertheless, the detection of zoonotic *Bartonella* species circulating in companion animals highlights the need for increased surveillance and greater awareness, as human infections may be underrecognized or misdiagnosed.

To the best of our knowledge, no pan-genome-based genotyping studies of *B. henselae* have been conducted in Central America. Our pan-genome analysis showed that *B. henselae* strains from Costa Rica did not cluster geographically with strains from a single country. Genetic diversity was also observed among Costa Rican strains, in contrast to findings from Chile, where local strains formed a single cluster (Banerjee et al., 2020). Comparative pan-genome analysis provides a useful framework for expanding the knowledge of shared and variable gene content within the genus *Bartonella*.

Comparison of allelic profiles between Costa Rican *B. henselae* isolates and global data identified ST1 and ST5, both of which have been previously reported (Arvand et al., 2007; Dias et al., 2023). ST1, a predominant variant in Asia and previously reported in Guatemala, Brazil, Argentina, Chile, and the United States (Arvand et al., 2007; Cicuttin et al., 2014; Bai et al., 2015; Dias et al., 2023; Sepulveda et al., 2023), matched the profile detected in a part of the studied feline population. ST5 is commonly found in Europe (Gil et al., 2013) and has been previously reported in Guatemala, Brazil, Chile, Argentina, and the United States (Arvand et al., 2007; Cicuttin et al., 2014; Dias et al., 2023; Sepulveda et al., 2023; Das Neves et al., 2025). The ST1 and ST5 variants detected in this study have been previously detected in Guatemala, indicating that these variants have already been established in Central America. Moreover, they have previously been associated with human diseases, supporting the potential relevance of companion animals as sources of human exposure (Arvand et al., 2007; Bouchouicha et al., 2009; Chaloner et al., 2011; Gil et al., 2013). The identification of sequence types circulating within this region emphasizes the importance of expanding genomic surveillance in Central America, where molecular epidemiological data on *B. henselae* remain limited. Continued characterization of local isolates is essential for determining the evolutionary origin, distribution, and epidemiological significance of this genotype. Broader data availability is also necessary to support reliable associations and robust classification of emerging *Bartonella* clones.

## Conclusion

To our knowledge, this is the first report of the isolation of zoonotic *Bartonella* spp. from companion animals in Costa Rica. These findings highlight the potential for human exposure to *Bartonella* spp. in settings where people live in close contact with companion animals. Because cats and dogs may harbor zoonotic *Bartonella* species and are frequently exposed to ectoparasites, infected animals and their fleas may contribute to bacterial circulation and represent potential sources of human exposure. Our results underscore the importance of flea control and support a One Health approach that integrates veterinary and human health surveillance to better understand the epidemiology and zoonotic potential of *Bartonella* spp.

This study documents both molecular detection and culture-based isolation of *Bartonella* spp. from domestic animals in Costa Rica and identifies circulating *B. henselae* sequence types ST1 and ST5. These findings contribute to our understanding of the genetic diversity of *Bartonella* and its evolutionary relationships in Costa Rica and the broader Central American region. The isolation of *B. henselae*, a recognized human pathogen, from companion animals highlights the need for further investigation of the ecology, transmission dynamics, and public health relevance of *Bartonella* spp. in the region.

## Data Availability

The datasets presented in this study can be found in online repositories. The names of the repository/repositories and accession number(s) can be found at: https://www.ebi.ac.uk/ena, PRJEB111650.

## Footnotes

1
https://www.ub.edu/dnasp/

2
https://itol.embl.de/

3
https://pubmlst.org/organisms/bartonella-henselae

4
https://cge.cbs.dtu.dk/services/MLST/

5
https://pathogen.watch/

6
http://www.mgc.ac.cn/VFs/

7 https://www.ncbi.nlm.nih.gov/datasets/genome/?taxon=38,323; accessed March 25, 2025.
